# Pathway analysis through mutual information

**DOI:** 10.1093/bioinformatics/btad776

**Published:** 2024-01-09

**Authors:** Gustavo S Jeuken, Lukas Käll

**Affiliations:** Science for Life Laboratory, KTH – Royal Institute of Technology, Stockholm 171 65, Sweden; Computer Science Department, Vrije Universiteit Amsterdam, Amsterdam 1081 HV, The Netherlands; Science for Life Laboratory, KTH – Royal Institute of Technology, Stockholm 171 65, Sweden

## Abstract

**Motivation:**

In pathway analysis, we aim to establish a connection between the activity of a particular biological pathway and a difference in phenotype. There are many available methods to perform pathway analysis, many of them rely on an upstream differential expression analysis, and many model the relations between the abundances of the analytes in a pathway as linear relationships.

**Results:**

Here, we propose a new method for pathway analysis, MIPath, that relies on information theoretical principles and, therefore, does not model the association between pathway activity and phenotype, resulting in relatively few assumptions. For this, we construct a graph of the data points for each pathway using a nearest-neighbor approach and score the association between the structure of this graph and the phenotype of these same samples using Mutual Information while adjusting for the effects of random chance in each score. The initial nearest neighbor approach evades individual gene-level comparisons, hence making the method scalable and less vulnerable to missing values. These properties make our method particularly useful for single-cell data. We benchmarked our method on several single-cell datasets, comparing it to established and new methods, and found that it produces robust, reproducible, and meaningful scores.

**Availability and implementation:**

Source code is available at https://github.com/statisticalbiotechnology/mipath, or through Python Package Index as “mipathway.”

## 1 Introduction

When conducting pathway analysis, we quantify the association between activity in a biological pathway and an alteration in phenotype, with the aim of providing a better overview of the large gene lists that typically are generated from high-throughput biological experiments.

Traditional methods to conduct such analysis first score each gene’s association between its expression and phenotype, then check for the enrichment of genes belonging to any pathway among the highest scoring genes (Dennis *et al.* 2003, Subramanian *et al.* 2005, Tian *et al.* 2005, Jeuken and Käll 2018). Such methods require a precomputed statistic as an input that quantifies its difference in expression between experimental conditions, and these statistics are typically a result of a linear model (Soneson and Delorenzi 2013, Love *et al.* 2014).

There are also topology-based pathway analysis methods that take a more mechanistic view of pathways. However, as they require complete annotation of, not just the participating analytes but also the way they interact, they are often seen as hard to use (Ihnatova *et al.* 2018), and require a more complete pathway annotation, that is often not present. A more recent alternative is to first quantify the activity of a pathway in each sample, and then score its association to the phenotype (Tomfohr *et al.* 2005, Barbie *et al.* 2009, Drier *et al.* 2013, Foroutan *et al.* 2018, Jeuken *et al.* 2022).

Most pathway analysis methods were originally developed for bulk data, yet some methods that rely on gene-level differential expression statistics are found robust also for single-cell data (Holland *et al.* 2020, Ma *et al.* 2020). However, obtaining reliable gene-level statistics for single cells is not trivial due to the presence of dropouts in the data (Kharchenko *et al.* 2014), with the two most common solutions being imputing the missing data, thus introducing a systematic bias, or simply ignoring this source of noise.

Relatively recently, other methods, such as SCPA (Bibby *et al.* 2022), were developed directly with single-cell data in mind. Yet, a restrictive technical limitation for the analysis remains: the size of the data generated by scRNA-seq experiments, often containing hundreds of thousands of data points, which brings memory and computational challenges that methods developed to work with bulk data are not equipped to face. Even methods explicitly developed for single-cell data, such as SCPA, resort to subsampling the data to make its size more manageable.

Here, we describe MIPath, a pathway analysis method that defines a pathway-specific random variable over any assay, although especially suited for single-cell data because it does this by clustering cells, which are more robust to dropout noise (Hou *et al.* 2020). It then tests its association with any phenotypical classification over the data. We use concepts from information theory that allow us to score this relationship without making many assumptions about the nature of the regulation of pathways.

## 2 Materials and methods

In this section, we use the word sample to refer to any data point that can have its transcription profile measured, such as tissues or single cells.

Our method, MIPath, consists of several interlinked steps. We (i) construct a Nearest Neighbor graph from the expression values in a pathway, then (ii) use those graphs to separate the samples into modules using a module detection algorithm. Subsequently, (iii) the adjusted mutual information between the phenotype and the modules is calculated. Each of these steps is described in detail in the subsections below.

### 2.1 Nearest neighbor graph

A *k* nearest neighbor (NN) graph can be constructed from any multivariate data by connecting elements *i* and *j* with a directed edge if *j* is one of the *k* points with the smallest distance to *i*. In our method, we will use Euclidean distances. Constructing such a graph can be computationally expensive if a large number of data points are involved, but the estimation of the approximate *k* nearest neighbors can be done much more efficiently (Dong *et al.* 2011).

Once the graph is constructed, we can add informative features such as edge weights. A similarity measure based on the number of shared nearest neighbors (SNN) has been shown to perform better than traditional similarity measures in high dimensional spaces (Houle *et al.* 2010) and this approach has already been shown to work well for gene expression (Levine *et al.* 2015). Conveniently, the number of shared neighbors between nodes can be efficiently calculated using a known property of adjacency matrices (Pemmaraju and Skiena 1990, p. 229). If *A* is the adjacency matrix of the directed NN graph, then $A^{T}$ is the same graph with the direction of edges reversed, and so $B=A\cdot A^{T}$ gives us an adjacency matrix from where we can extract the SNN information, that is because $B_{ij}$ is the number two-step paths nodes *i* and *j*, the first taken in *A* and the second in $A^{T}$. This is the same as the number of shared neighbors of *i* and *j* in *A*. To convert the number of shared neighbors to a similarity metric, we must divide this number by the total number of neighbors of *i*, which is exactly *k*.

Practically, starting with a data matrix (e.g. gene expression) for each pathway, we construct a graph of data points. Here, each vertex represents a data point on the original data, such as cells or bulk samples, and they connect to data points with very similar expressions for the given pathway. This graph then represents the local patterns for the given pathway in the original data.

### 2.2 Leiden algorithm for module detection

If the nodes of a graph are separated into groups, its modularity is defined as the difference between the fraction of edges that fall within the groups and the expected fraction of these that are expected with a random distribution of edges. More specifically, it can be measured as

$$Q=\frac{1}{2m}\sum_{ab}\left[A_{ab}-\frac{k_{a}k_{b}}{2m}\right]\delta (c_{a},c_{b})$$

where *m* is the number of nodes, $A_{ab}$ is an indicator variable of the existence of an edge between nodes *a* and *b*, $k_{a}$ is the degree of node *a* and $\delta (c_{a},c_{b})$ is another indicator for the group assignment of nodes *a* and *b* ($c_{a}$ and $c_{b}$) being the same (Barabási and Pósfai 2016). This can be adapted to deal with edge weights by changing $A_{ab}$ to the weight of the edge from *a* to *b*, and to a directed network by changing $k_{a}$ and $k_{b}$ to $k_{a}^{in}$ and $k_{b}^{\mathrm{out}}$, the in-degree and out-degree of nodes *a* and *b* respectively, and then multiplying the score by 2 (Leicht and Newman 2008).

The problem separating the nodes of a graph into modules in an optimal way can be viewed as an optimization problem on *Q*, with respect to the group assignments $\left\{c_{a},c_{b},\ldots \right\}$. The solution to this problem is unfortunately not trivial. The Louvain method (Blondel *et al.* 2008) first introduced a greedy approach to this problem that can be computed apparently in log-linear time (Lancichinetti and Fortunato 2009). The Leiden algorithm (Traag *et al.* 2019) improves on this method by providing further guarantees with a faster running time. The Leiden algorithm has also been used successfully by the Seurat package (Stuart *et al.* 2019b) on SNN networks, to find subpopulations of cells on single-cell transcriptomics data.

We use this on the graphs generated for each pathway, finding groups of data points where the pathway activity is very similar. For single-cell datasets, this would be analogous to finding subpopulations when only considering the gene expression information from one specific pathway, with the main assumption then being that cells in each subpopulation have a similar activity for this specific pathway. An important consequence of using clusters as the source of signal for the subsequent analysis is that these are more robust to dropout noise present in single-cell datasets, and as such the same idea is, for instance, used to find subpopulations of cell types in large datasets (Butler *et al.* 2018).

### 2.3 Adjusted mutual information score

Mutual information (MI) measures how much two random variables are associated with each other. More precisely, it measures the amount of information that is obtained about one of the variables when measuring the other. Importantly, it does not model the nature of the interaction between those variables, as it only looks at each variable’s entropy and their joint entropy. It is then an extremely useful measure of dependence where the shape of the interaction is not known or can take multiple forms. We believe that this makes it ideal for the task of finding associations between pathway activity and phenotype, as they can be very hard to model in general and also have different shapes for each pathway analyzed.

The MI of two random variables *X* and *Y* can be calculated as the Kullback–Leibler (KL) divergence (also known as relative entropy) between the joint distribution Pr(X,Y) and the product of the marginals Pr(X)Pr(Y):

(1) $$\text{MI}(X,Y)=D_{\text{KL}}\left(\mathrm{Pr}(X,Y)||\mathrm{Pr}(X)\mathrm{Pr}(Y)\right)$$

Another form of this expression is:

MI(X,Y)=H(X)+H(Y)−H(X,Y)

where H(X) is the entropy of a random variable *X*. This gives us a more intuitive interpretation of this measure. We can see that if the joint entropy is the same as the sum of the entropy of both variables, the MI is zero. Also, by using the chain rule, we can see that the MI is maximal if the conditional entropies H(X|Y) or H(Y|X) are zero.

In our method, we will use MI to study the association between two partitions of a dataset, one that is based on gene expression, and another based on phenotype. As a metric, MI generally gives values in the range [0,+∞], which makes comparing these results difficult. However, it can be shown that the maximum MI between partitions *X* and *Y* is $\frac{1}{2}\left(H(X)+H(Y)\right)$ (Romano *et al.* 2016). We can then define a Normalized Mutual Information (NMI) measure as:

$$\text{NMI}(X,Y)=\frac{\text{MI}(X,Y)}{\max\text{MI}(X,Y)}=\frac{\text{MI}(X,Y)}{\frac{1}{2}\left(H(X)+H(Y)\right)}$$

which is bounded to [0, 1], making comparisons easier. There is yet another problem in comparing NMI metrics, this time on the lower bound, because, by random chance, MI will be higher when we have a bigger number of partitions (Vinh *et al.* 2010). We then need to define another measure, Adjusted Mutual Information (AMI), that takes into account this variation of the baseline:

(2) $$\text{AMI}(X,Y)=\frac{\text{MI}(X,Y)-E[\text{MI}(X,Y)]}{\max\text{MI}(X,Y)-E[\text{MI}(X,Y)]}$$

where E[MI(X,Y)] is the expected MI when both partitions *X* and *Y* are random. AMI will therefore be 1 if MI(X,Y)=maxMI(X,Y) and 0 if MI(X,Y)=E[MI(X,Y)], and can also take very small negative values. This will be the measure used throughout this study.

Calculating E[MI(X,Y)] can be hard. However, here we have an advantage if using discrete classes, as by random the distribution of data on those classes will follow a generalized hypergeometric distribution. Vinh *et al.* (2010) shows us how to calculate E[MI(X,Y)] under this model of randomness.

Practically, we use AMI to compare the partition of data points (e.g. subpopulations) we found in the results of the Leiden algorithm with the partition of data points given by any phenotype annotation. Following our initial assumption for these populations, we are then testing. for each pathway, the association of different regulation regimes for a pathway, with different phenotypes.

### 2.4 Adjusted conditional mutual information scoring

It is also possible to calculate the conditional mutual information MI(X,Y|Z), where *Z* is a third random variable. This is done simply by substituting the joint Pr(X,Y) by Pr(X,Y|Z) as well as the marginals Pr(X) and Pr(Y) by Pr(X|Z) and Pr(Y|Z), i.e. this can be calculated using the KL divergence as:

$$\text{MI}(X,Y|Z)=D_{\text{KL}}\left(\mathrm{Pr}(X,Y|Z)||\mathrm{Pr}(X|Z)\mathrm{Pr}(Y|Z)\right)$$

For a discrete variable Z∈Z, this can also be expressed as:

(3) $$\text{MI}(X,Y|Z)=\sum_{z\in Z}p(Z=z)D_{\text{KL}}\left(\mathrm{Pr}(X,Y|z)||\mathrm{Pr}(X|z)\mathrm{Pr}(Y|z)\right)$$

We can also define an AMI measure for the conditional probabilities:

$$\text{AMI}(X,Y|Z)=\frac{\text{MI}(X,Y|Z)-E[\text{MI}(X,Y|Z)]}{\max\text{MI}(X,Y|Z)-E[\text{MI}(X,Y|Z)]}$$

Where $\max\text{MI}(X,Y|Z)=\frac{1}{2}\left(H(X|Z)+H(Y|Z)\right)$ (following the above) and the calculation of E[MI(X,Y|Z)] can be found in the Supplementary Information.

To interpret the results of AMI(X,Y|Z), it is useful to compare it to AMI(X,Y). By looking at Equations (1) and (3) and the normalization that follows, we can see that situations where AMI(X,Y|Z)<AMI(X,Y) are those when for each value of *Z* the association of *X* and *Y* is weaker on average than AMI(X,Y), where we have no knowledge of the variable *Z*, and in this case *Z* can be viewed as a confounder.

### 2.5 Target pathways

Benchmarking the performance of a pathway analysis method is not a trivial task. For evident reasons, it is frequently done by interpreting the resulting pathway scores in the context of the experiment that was performed, as this is usually the circumstance on which these methods are required to perform. However important, this is a subjective measure of performance, where methods can be seen as successful both for presenting sound and expected results or for elucidating new mechanisms.

Nguyen *et al.* (2019) argues for a reproducible measure for the success of pathway analysis methods. By selecting datasets with conditions that already have an associated pathway, e.g. Parkinson’s disease, one can benchmark methods by how they rank said pathway in their results. Bias can be avoided by selecting the datasets and target pathways before running any experiment. Here, we selected single-cell datasets that contained cells taken from diseased, as well as normal tissues, and compared these two groups. We then benchmark methods by how high the respective disease pathway is scored.

We would like to note that this measure is very reductionist. The usefulness of a pathway analysis method should be evaluated by how well it can help elucidate the molecular processes that are associated with the experiment being analyzed. Again, a good pathway analysis method will both help to describe these processes, as well as provide new hypothesis to be tested. These extensive analyses, however, do not make for a reproducible and fair way to benchmark methods, as they are subjective and thus can be very prone to confirmation bias. In this article, we will first use target pathways to that our method is competitive with established methods, and later provide more in-depth analysis of some datasets, to show its usefulness.

### 2.6 Datasets

Most datasets were downloaded from the Single Cell Expression Atlas (Papatheodorou *et al.* 2020). Their ascension number, associated publication, target pathway, and size can be found in Table 1. For each dataset, we have selected a target pathway following the criteria mentioned in the previous section. All these datasets were collected to study the effects of specific disease, and contain both normal and diseased cells. We then select the specific disease pathway as the most likely to be different between these sample groups. These were selected before any analysis were done.

**Table 1.** The Single Cell Expression Atlas datasets used in the comparison.^a^

Dataset	Accession	Publication	Target pathway	Cells
1	E-MTAB-10290	Strnadová *et al*. (2022)	Melanoma	29 203
2	E-MTAB-11011	Schultheiß *et al*. (2021)	COVID-19	11 084
3	E-MTAB-8322	Alivernini *et al*. (2020)	Rheumatoid arthritis	70 246
4	E-MTAB-8884	Wiseman *et al*. (2020)	Chronic myeloid leukemia	8281
5	E-CURD-97	Miragaia *et al*. (2019)	Colorectal cancer	1908
6	E-HCAD-31	Fang *et al*. (2019)	Type II diabetes mellitus	18 694
7	E-HCAD-36	Li *et al*. (2020)	Type II diabetes mellitus	58 407
8	E-MTAB-9221	Silvin *et al*. (2020)	COVID-19	6807
9	E-GEOD-149689	Lee *et al*. (2020)	COVID-19	70 553
10	E-GEOD-150728	Wilk *et al*. (2020)	COVID-19	57 096
11	E-ENAD-27	Lawlor *et al*. (2017)	Type II diabetes mellitus	1157
12	E-GEOD-83139	Wang *et al*. (2016)	Type II diabetes mellitus	635
13	E-GEOD-76312	Giustacchini *et al*. (2017)	Chronic myeloid leukemia	2151
14	E-MTAB-7303	Lang *et al*. (2019)	Parkinson disease	568
15	E-GEOD-81608	Xin *et al*. (2016)	Type II diabetes mellitus	1600
16	E-MTAB-5061	Segerstolpe *et al*. (2016)	Type II diabetes mellitus	3514

a Each dataset is listed with the phenotype variable we used for testing and the pathway we selected as a target pathway.

The METABRIC dataset (Curtis *et al.* 2012), was downloaded from European Genome-Phenome Archive under accession number EGAS00000000083.

As a source of pathway annotation, we have used the KEGG (Kanehisa and Goto 2000) pathways, and Reactome (Gillespie *et al.* 2022) pathways downloaded from the project’s website. Reactome pathways were preferred when performing analysis due to their granularity when compared to KEGG pathways. However, when benchmarking methods using target pathways, we use KEGG pathways because the database supplies us with pathways with systemic responses for many diseases.

## 3 Results

### 3.1 A pathway analysis method based on mutual information

For each pathway, we start by calculating the *k* approximate nearest neighbors of each sample, following Dong *et al.* (2011), and using a Euclidean distance metric on the normalized genes. Here, the distance metric used involves only the genes present on the pathway of interest, thus capturing only the sample structure present in the subspace defined by this pathway. This results in a directed graph that is then weighted using the SNN similarity.

At this point, we have a collection of graphs, one for each pathway, that have the exact same number of nodes and edges, with the only difference being that each is wired to represent neighborhoods on different pathway subspaces. To these, we apply the Leiden algorithm to detect modules present, and the resulting module assignments of the samples is used as a representation of each sample state for the pathway. Since the number of modules is a result of the algorithm, a pathway may have any number of states.

**Figure 1.**
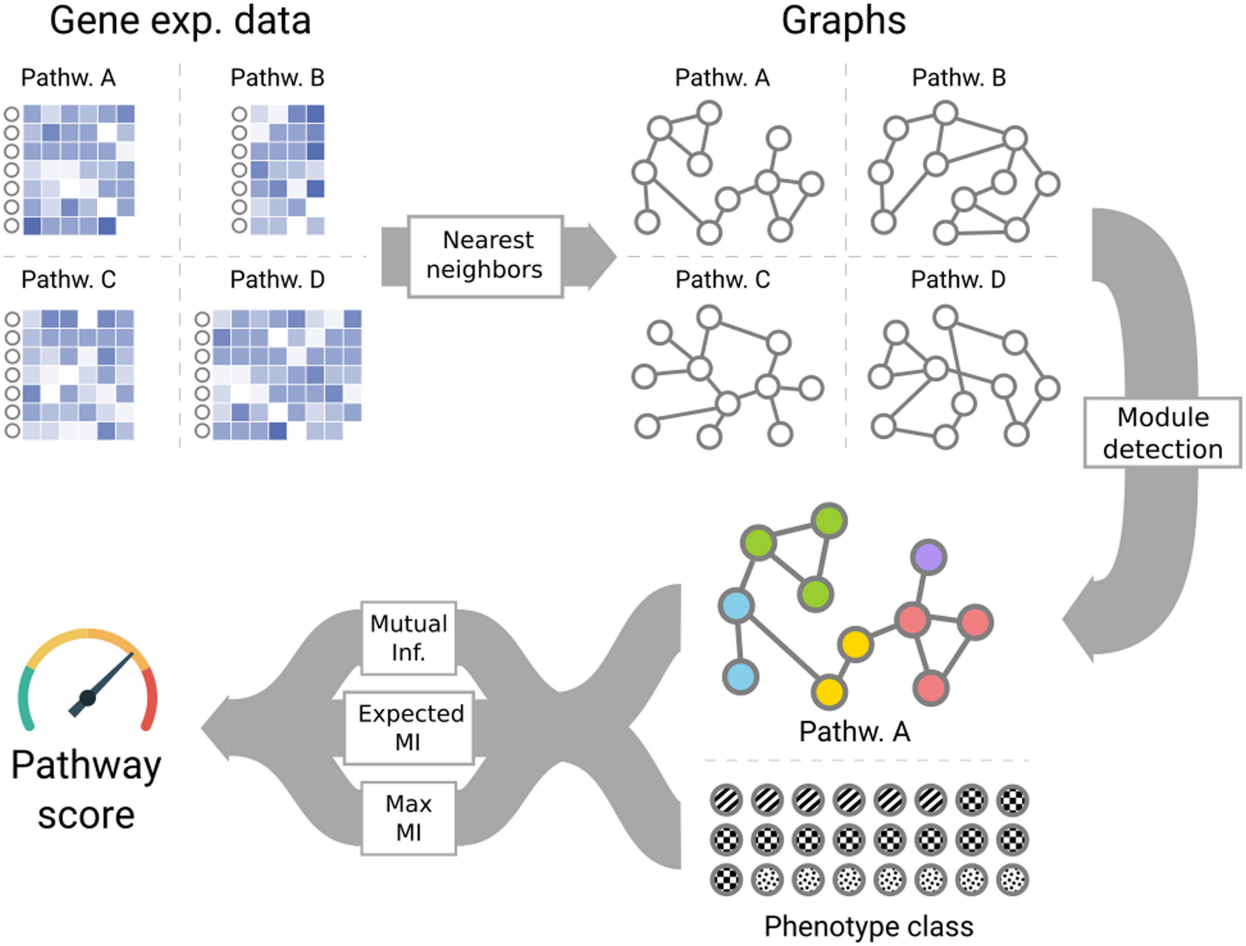
An illustration of the steps involved in the MIPath method. The expression matrix for each pathway is converted into a graph using a nearest neighbor approach. The graphs are then used to partition the samples into modules. Finally, adjusted mutual information is used to score the relationship between the sample phenotypes and their partition in the graph.

The third step produces the pathway score by calculating the Adjusted Mutual Information between this pathway state of each sample and any other sample-specific variable, such as annotations of phenotypes. Here we note that we are not limited to studying case and control, but can score any discrete variable. We also see here the importance of correcting this measure for random chance: The expected mutual information at random is directly affected by the number of clusters produced by the previous step (Vinh *et al.* 2010), so this correction is essential if we want to compare scores between different pathways. Figure 1 presents an overview of the method.

The resulting process has several methodological advantages, the first is that it has few strong assumptions on how gene products interact within a pathway, and how these interactions result in a change of phenotype. MIPath uses mutual information as the basis for its score, and thus it can quantify the dependence between genotype and phenotype using only the shape of their (non-parametric) joint distribution. Most of the established pathway analysis methods rely first on differential expression analysis, which has its own set of assumptions on what it means for gene products to be differentially regulated. They then combine the numerical results of these analyses, often in the form of a statistical significance score, into a single pathway score, in the process making assumptions on how they interact with each other. MIPath produces pathway scores directly from the gene expression data, with the main simplifying assumption being that samples cluster together differently when different sets of genes are used.

Secondly, we note that there is only one important parameter in the method, the *k* number of neighbors, that presents a trade-off between local and global information. However, the importance of this parameter is diminished by the use of the SNN similarity (Houle *et al.* 2010), and in the next section, we show that it cannot be used to fine-tune its results. This is desirable from a user point of view, as the meaning of a model parameter is not always obvious. In theory, setting the correct value for a parameter often involves understanding the model and underlying data. Practically, it is commonly done by either using the default values provided or using the ones which produce the most desirable results, which again is prone to bias. By developing a model with few parameters, we hope to lessen this burden on the user.

Finally, the method is fast. It can analyze a dataset of 50 000 cells for each of 337 KEGG pathways in under 1.5 h, using a simple quad-core laptop with 16 GB of RAM. To compare this performance to another method developed for single-cell data, SCPA (Bibby *et al.* 2022), we use a smaller dataset of 1908 cells (Miragaia *et al.* 2019), a fairer comparison due to SCPA downsampling strategy. MIPath here runs the full analysis in 2.5 min, in contrast to 6.8 min taken by SCPA, even if SCPA downsamples the dataset to 1000 cells.

### 3.2 Effects of parameter *k*

As stated, *k* is the only parameter of the model. The approximate nearest neighbor algorithm used has an empirical complexity of $O(n^{1.14})$ on the number of samples (Dong *et al.* 2011), and the Leiden algorithm appears to run in O(n log n) on the number of edges of the graph, which is directly related to k×n. Thus, *k* has an effect both on the performance and the runtime of the method.

**Figure 2.**
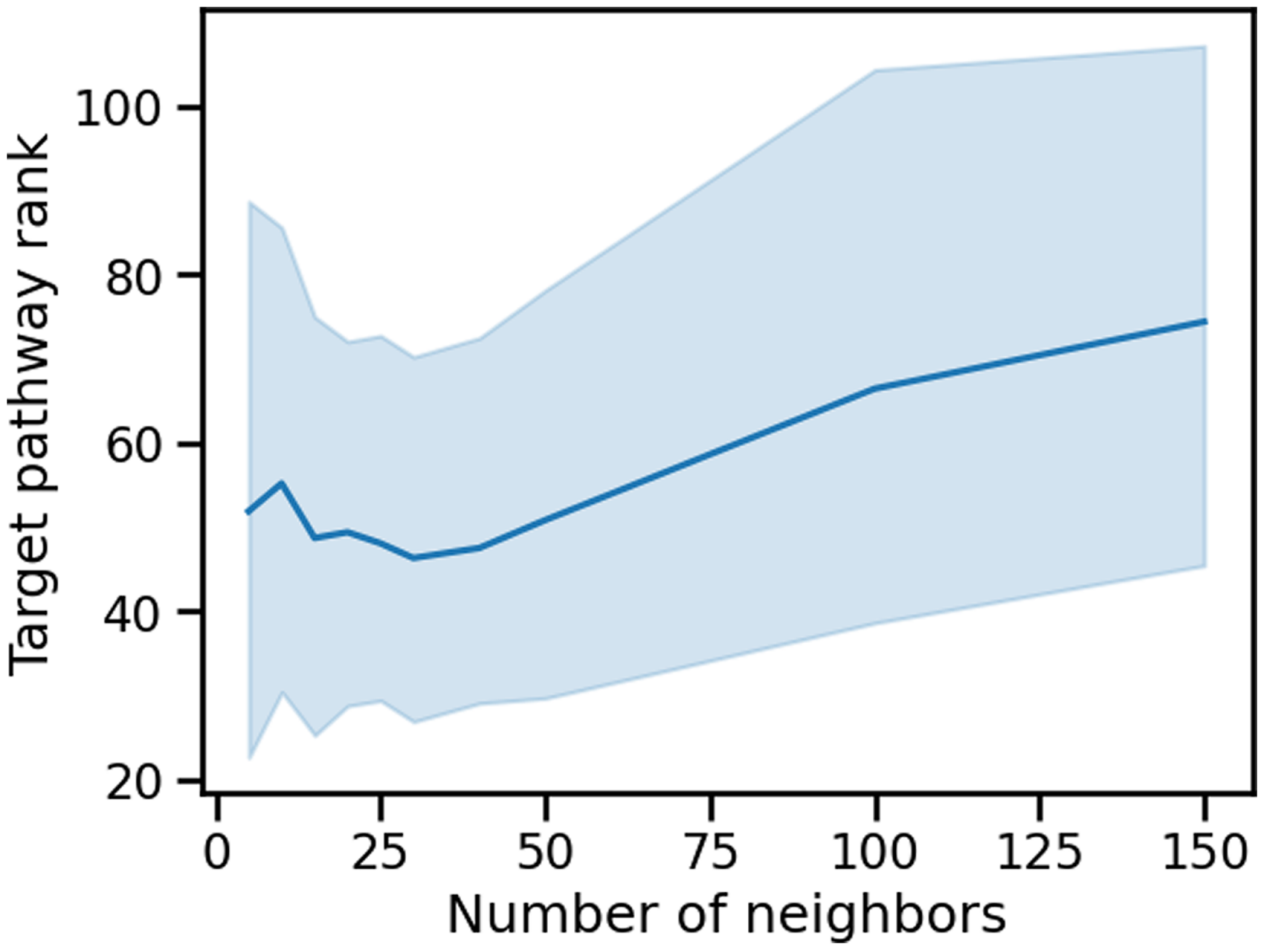
The effect of the selected number of nearest neighbors, *k*, on the method’s performance. We plotted the aggregated target pathway’s rank performance (of all datasets in Table 1) for different values of *k*.

To test the actual effects of *k* on the performance of the method, we have applied it to the datasets on Table 1 with varying k∈{5,10,15,20,25,30,40,50,100,150}. Figure 2 shows an aggregate view of how well the target pathways of each dataset ranked.

We see the best performance of our method in the range of [25, 40]. To the right of this range, the performance loss is dominated by the datasets with fewer samples because if *k* is larger than (or approaching) the cluster size, the nearest neighbor graph fails to capture global cluster information accurately. To the left of this range, the loss of performance results from less local information on the graphs.

As a compromise between performance and runtime, we selected k=25 as the method’s default value. Unless otherwise specified, we here use k=25 for all our further results.

### 3.3 MIPath captures a meaningful signal

We want to check if the method can capture a meaningful and stable signal, and one way we can do this is to test if the results are reproducible. To simulate multiple experiments under the same conditions, we can subsample a dataset multiple time, each time performing our method on this reduced dataset, and see how comparable the resulting scores are.

**Figure 3.**
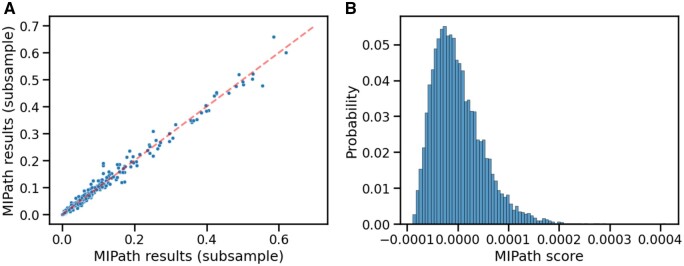
(A) Correlation between the results of two subsamples. We plot the MIPath score of a subsample of the dataset in Table 1, against the scores for a second subsample. The dashed line indicates equal scores. We see high concordance between the results. (B) Example of empirical null distribution obtained by permuting the associations between cells and phenotype, 10 000 times. By looking at the scale, we see how small the variance of the null distribution.

Practically, for every dataset on Table 1 with over 10 000 samples, we have subsampled 20% of the dataset 10 times, and we scored the association between KEGG pathways and the tested phenotype for each subsample with our method. Having then ten sets of scores, we calculated the Pearson’s correlation between every two pairs of sets to get an average correlation over all subsamples. We have observed that the results between runs closely align, with the average pairwise Pearson’s correlation between subsamples of the same dataset being very high at 0.96. Figure 3A shows an example of how two random subsamples of the datasets relate to each other.

### 3.4 MIPath is very sensitive

Through the same approach used to calculate the expected value of mutual information at random, we can calculate the variance by:

$$\text{Var}(\text{MI})=E[{\text{MI}}^{2}]-E{\left[\text{MI}\right]}^{2}$$

Which allows us to approximate a null distribution for the statistic. In practice, however, calculating $E[{\text{MI}}^{2}]$ involves a large sum over many small values, which is numerically difficult to do precisely. Fortunately, the most computationally expensive part of the method is not the scoring, which allows us to precompute the partition of samples first and then perform a sample label permutation, each time calculating the resulting score, and generate a precise empirical null distribution for the score. Figure 3B shows an example distribution. On Table 2 we calculate *P* values for MIPath using 10 000 such permutations, and estimate de FDR following Storey and Tibshirani (2003).

**Table 2.** A comparison of the performance between MIPath, GSEA, Enrichr, and SCPA, based on the rank of selected target pathways for different datasets.^a^

Dataset	MIPath	GSEA	Enrichr	SCPA
1	138	228.5*	94	164.5
2	41	177*	12.5	32
3	24	171.5*	15.5	14.5
4	100	170.5*	155*	118
5	177	170.5*	89	118
6	33	82*	143	11
7	9	148*	140	6
8	21	309.5*	18	21.5
8	54	208*	23.5	38
10	34	189.5*	37	8
11	16	145*	3	9.5
12	7	183.5*	17	10
13	65	171.5*	101	161
14	13	251.5*	1	12*
15	9	172*	20	10
16	16	78*	182	193
Mean	47.31	178.53	65.71	57.93
SD	50.02	56.41	62.39	67.41

a For the comparison, we used the KEGG pathway database with 337 pathways. If two pathways had the same score, their ranks were averaged. An asterisk indicates that the target pathway did not pass the significance threshold of FDR=0.05 according to the method. Mean and standard deviations are shown to summarize the results.

Both approaches result in a very small variance in the null distribution for a sufficiently large number of samples, which is expected given the combinatoric nature of the null model. The result of this is that most non-zero MIPath scores are significant, even if the effect itself is small.

### 3.5 MIPath successfully identifies target pathways and benchmarks well against other pathway analysis methods

As a benchmark, we compare our methods to GSEA (Subramanian *et al.* 2005), Enrichr (Xie *et al.* 2021) and SCPA (Bibby *et al.* 2022), two widely used methods of pathway analysis, and a new one designed specifically for single-cell pathway analysis, on the datasets in Table 1. Since these are all single-cell datasets that contain cells from both normal and diseased tissue, a good assumption is that the KEGG pathways of the respective diseases are some of the most differentially regulated between these two groups. Thus, we benchmark the methods by their ability to retrieve this information, as explained in the methods section.

For each dataset, we run our method scoring each pathway using the sample annotation provided, and we rank the results by their MIPath score.

GSEA and Enrichr require a previous statistical analysis to establish differential expression of genes between groups. Since we are allowing more than one group, we perform a one-way ANOVA for each gene and get a significance value for the differential expression of the gene. This ranked list of *P* values is used as an input to GSEA. The method calculates an enrichment score and compares it to 10 000 random permutations to obtain a *P* value for each pathway. The results are then ranked by their *P* value.

Enrichr needs as an input a set of genes, rather than a ranked list, so we need to divide the list into differentially and not differentially expressed genes. For this, we first control for multiple testing by converting the *P* values into *q* values following Storey and Tibshirani (2003), and threshold the list at a *q* value of 0.05. The set of differentially expressed genes is imputed into Enrichr and its results are ranked by *P* value.

SCPA takes as an input two groups of cells, and so we compare the normal cells against all others. The pathway results are then ranked by their *P* value.

Table 2 shows the results of the benchmark, and we see that our method has the lowest average rank for the target pathways. We also have the lowest standard deviation on the ranks. We note that no target pathway fell below the .05 *P* value threshold with GSEA.

Note that we in several of the datasets made naïve assumptions about their nature. Post-analysis, we tend to examine the underlying studies more carefully and conclude that other pathways would be more representative of the actual biological effect in the study. In any such case, we purposely selected to not change our minds about which pathway should be the target pathway, as we otherwise would be likely to bias the test.

### 3.6 Analysis of hematopoietic cells dataset

We now investigate the ability of our method to test multiple sample groups at the same time, such as cell types. For this, we use a dataset that consists of more than 8000 cells from the hematopoietic cells partitioned into 23 cell types (Ranzoni *et al.* 2021), scoring all KEGG pathways based on their association with this partition. Here the KEGG pathway “Hematopoietic cell lineage” was found to have the largest MIPath score and hence the major differentiating pathway. The second most differentiating pathway was “Cell adhesion molecules (CAMs),” which indeed should be a differentiating factor between the different cell types in the immune system, e.g. helper T-cells and B-cells. The third most important pathway was “Cytokine-cytokine receptor interaction,” which is yet another pathway involved in the immune system, and hence differentiated over the different cell types in the hematopoietic cell lineage. This can be contrasted with the top-ranked pathways of GSEA, which are “Pantothenate and CoA biosynthesis,” “Asthma,” and “*Escherichia coli* infection,” which all are less obviously associated with the difference in cell functions of the hematopoietic cell linage.

**Figure 4.**
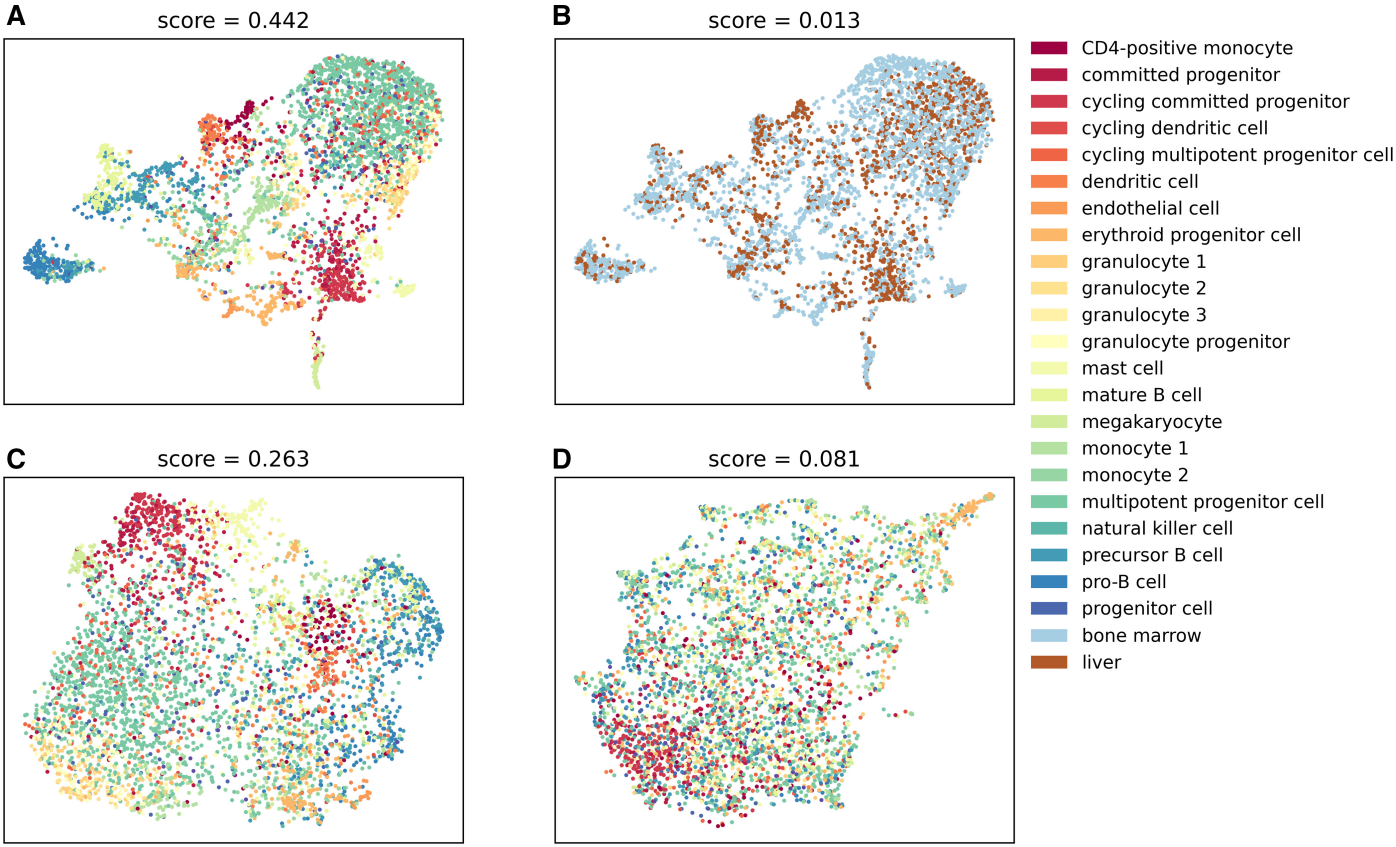
High-scoring pathways indicate a better separation of the samples. We made UMAP plots of the distribution of samples in dataset 7 according to three pathways, above each plot is the MIPath score for the association. (A) and (B) The “Hematopoietic cell lineage” pathway, colored by cell type (A), with good separation and high score, and colored by sample origin, bone marrow, or liver (B), showing a low score and bad separation. (C) “JAK-STAT signaling” pathway and (D) “Lysine degradation” pathway are also shown as an illustration of how increasingly worse separation will result in lower scores.

Figure 4 provides an intuitive illustration of the MIPath scores. We have made UMAP plots from the dataset using only genes from specific pathways to illustrate how well the cell types separate on those spaces. Figure 4A shows the plot for the top scoring pathway “Hematopoietic cell lineage,” we see that the cell types are clearly separated by the genes in this pathway, but using the same pathway we cannot separate well different phenotypes such as the part of the body the cells were taken (Fig. 4B). Figure 4C and D shows how the separation of cell types appears with lower-scoring pathways (“JAK-STAT signaling” and “Lysine degradation”) to illustrate how, with lower scores, the cell types start to blend. This illustration can also be seen for other datasets in Supplementary Fig. S1.

### 3.7 Analysis of interferon gene therapy mouse dataset

Escobar *et al.* (2018) have used gene therapy to induce over-expression of interferon-alpha (IFN-α) in mouse models. They have shown that mice that have undergone such therapy show increased antitumor properties if compared to a control.

We apply MIPath to this dataset, first checking for pathways that are associated with therapy versus control phenotype. Here, to have a deeper understanding of the response process, we used the Reactome pathways due to their better granularity when compared to KEGG. In the results, we see that four out of the five pathways that have the strongest association with this phenotype involve RHO GTPase signaling and its effectors, which are known to regulate many cell processes, such as proliferation. The most specific of these five pathways, “RHO GTPases Activate NADPH Oxidases,” elucidates how this signaling regulates known pro-tumorigenic molecules.

### 3.8 The method provides a natural way to deal with confounders

Since most gene expression experiments, especially in single cells, are observational, when studying the association between gene expression and phenotype one must always be aware of the possible presence of confounders. Confounding variables affect both gene expression and phenotype, causing a spurious relationship between the two that may be interpreted as a causal relationship if not adequately studied.

Our method has a very straightforward way of dealing with confounders by using conditional distributions. By conditioning both marginals and the joint on a possible confounder, we are effectively stratifying the data, as shown by Equation (3), and thus remove the effect of such a confounder from our scores. It is important to note that this is not an add-on but instead an inherent property of the method, as we use a joint distribution to obtain a score. Conditional joint distributions are a natural way to model confounding variables in the association of two variables. Thus, we have a very robust way to model confounders in our method, with the drawback being that the use of conditional distributions results in less power with the same number of data points, as it is again analogous to stratification.

One of the clearest examples of confounders that applies to pathway analysis is the patient age in the context of survival analysis. Since the survival variable is measured as the time between measurement and the disease event, age explains much of the variance in this variable. Still, age also affects many biological processes in the cell. Thus, if one intends to study the effects of different pathways on the survival of patients with some disease, one must be aware of the spurious relationship between those two that the age of the patients might cause.

We tested how our model can handle this case on a breast cancer dataset. For this, we downloaded gene expression data from the METABRIC dataset, consisting of microarray reads from 1992 breast cancer specimens, together with the clinical annotation containing the survival information and age of the respective patients. We then classify the patients into five quantiles for both age and survival time. After standardizing the gene expression, we run our method to test the association between all Reactome pathways and the survival quantiles, as well as the same association when conditioning on the age quantiles.

If we rank the pathways with the largest difference between the unconditioned score and the ones where we use the conditional distributions on age, we can find the ones where the spurious relationship caused by age is strongest. Among the top 1% of pathways with the largest drop in score are: “Inhibition of nitric oxide production” (Torregrossa *et al.* 2011), “Defective Mismatch Repair Associated With MSH6” (Ryan *et al.* 2017), “Signaling by Erythropoietin” (Ershler *et al.* 2005), “ABC transporter disorders” (Efferth 2003), “Biosynthesis of protectins” (Das 2018) and “Elevation of cytosolic Ca2+ levels” (Raza *et al.* 2007), all of which have been shown to be associated with aging. Other well-known pathways related to aging, “Extension of Telomeres” (Aubert and Lansdorp 2008) and “Serotonin and melatonin biosynthesis” (Rozencwaig *et al.* 1987), are also among the top 3%. The two most significant pathways that saw a decrease in score were “Nucleotide Excision Repair” and “Signaling by NOTCH1 in Cancer,” which are associated with both cancer and ageing (Marteijn *et al.* 2014, Balistreri *et al.* 2016). We also observe that some of the most significant pathways, such as “Regulation of TP53 Activity” and “Extra-nuclear estrogen signaling,” have seen a slight increase in score after controlling for age.

Another simple example of how confounder can be managed by our method can be seen by analyzing the data from Stuart *et al.* (2019a), where they show many different routes with which a post-implantation epiblast stem cell (EpiSC) can be programmed into a naïve pluripotent stem cell. They submit EpiSC cells to three different treatments designed to induce different metabolic and signaling responses, which all converge to the same end state, with a control group not receiving any treatment. We use our method to find which pathways are differentially regulated on the different cell types of the dataset, and we find that several signaling pathways are the ones most strongly associated with this difference, especially Signaling by Receptor Tyrosine Kinases. Next, we condition our analysis on the type of treatment given to each cell and find that no signaling pathway is now significantly different, the results for the top 10 pathways can be seen on Supplementary Table S1. This is expected because the treatment type is a confounder that affects both gene expression and cell state, but most importantly, because every cell that was submitted to any of the three treatments is in the same state.

## 4 Conclusion

In this paper, we have presented a new method for pathway analysis, MIPath, that differs from others for its use of concepts in information theory to score the association between pathway activity and phenotype. Information theory as a field has long dealt with the problem of analyzing signals in complex systems, and as such is very suitable for applications in molecular biology (Chanda *et al.* 2020). Practically, we find modules of samples for each pathway and score the association between these modules and any phenotype classification by using a mutual information score that has been normalized and corrected for randomness.

This process has several advantages. First, it does not rely on any previous statistical analysis to rank or select significant genes, as it identifies sample modules directly from the gene expression. By using the subspace of gene expression defined by each pathway, and converting it to a nearest neighbor graph (with shared neighbor weights), we avoid performing the module detection step on spaces of different dimensionality. This is important as many clustering methods are known to have varying performance depending on the dimensionality of the space (Houle *et al.* 2010). This also makes the method more robust to dropout noise in single-cell data (Hou *et al.* 2020).

Second, by scoring using mutual information and embedding the hypergeometric error model in the calculation of each individual score, we get a very sensitive model that can detect even weak signals at high significance levels. This, combined with the score normalization, also make the scores comparable across experiments. An added benefit is the possibility of using conditional mutual information to deal with confounding factors.

Third, it has only one parameter, namely the number of neighbors used for the graph construction, which had an intuitive meaning as the tradeoff between the global and local structure of the samples. Practically, however, it does not have a big effect on the performance of the method, thanks in part to the use of the shared nearest neighbor’s similarity metric.

Lastly, this test is self-contained, meaning that the scores for each pathway depend only on the expression of genes belonging to that pathway, and do not rely on a gene expression background, which further increases its sensitivity (Goeman and Bühlmann 2007).

We have shown that this method captures a stable signal and that it performs well by measuring how highly known targeted pathways are ranked in 16 different datasets, and comparing it to the ranks obtained by GSEA, Enrichr and SCPA under the same conditions. We have also shown that conditional mutual information can successfully deal with confounders.

There are, however, some drawbacks to the model. The biggest is that it does not handle continuous variables, and variables such as survival or age have to be discretized into quantiles in order to be scored. This stems from the fact that we rely on empirical distributions to be able to calculate the mutual information. Even the gene expression itself is also discretized in a way, with samples that are close together in a pathway subspace considered equal, which results in a theoretical loss of information.

Another important limitation is that due to the use of the Leiden algorithm to find sample modules, our method requires more samples than others to produce a meaningful result. Further improvements could be made here, with alternate methods for finding the sample modules depending on the set size, or even allowing for more omics modalities to be included.

Overall, pathway analysis methods provide an extremely powerful tool for the analysis of omics data. They combine carefully curated theoretical knowledge of molecular biology in the form of pathways, with the data-driven approach of high throughput experiments. Yet, due to complex models and assumptions, the results of such analysis are not trivial to interpret. By providing a simple and robust method with no fine-tuning parameters, we hope we make this a more straightforward task for bioinformaticians.

## Supplementary Material

btad776_Supplementary_Data

## Data Availability

The source code for MIPath can be found at https://github.com/statisticalbiotechnology/mipath. It is also published in the Python Package Index and can be installed using “pip install mipathway.”
