# Supplementary material for: Pathway analysis through mutual information
Source: Bioinformatics. 2024 Jan 9;40(1):btad776. doi: 10.1093/bioinformatics/btad776 (PMC10783954; doi:10.1093/bioinformatics/btad776)
Supplement: btad776_Supplementary_Data [file btad776_supplementary_data.pdf]

Supplementary Information: Pathway Analysis Through Mutual Information

Gustavo S. Jeuken[1,2*] and Lukas Käll[1*]

[1]Science for Life Laboratory, KTH – Royal Institute of Technology
[2]Current address: Department of Computer Science, Vrije Universiteit Amsterdam
[*]Corresponding authors

Expected conditional mutual information

Nguyen et al.[1] shows us how to calculate the expected mutual information over two random partitions of the data using a hypergeometric model for randomness. To extend this result to the conditional mutual information, we start with the definition of $I(X, Y|Z)$ for the discrete variables X, Y and Z with support $\mathcal{X}$, $\mathcal{Y}$ and $\mathcal{Z}$ respectively:

$$I(X,Y|Z) = \sum_{z\in\mathcal{Z}} p_Z(z) \sum_{x\in\mathcal{X}} \sum_{y\in\mathcal{Y}} p_{X,Y|Z}(x,y|z) \log\left(\frac{p_{X,Y|Z}(x,y|z)}{p_{X|Z}(x|z)p_{Y|Z}(y|z)}\right) \quad (1)$$

and apply the same convention to its joint contingency table. We now have the tensor $M = [n_{ijk}]$ where $i = 1...R$, $j = 1...C$ and $k = 1...S$, $A = [a_{ik}]$ and $B = [b_{jk}]$ are the 2-way tables of (X, Z) and (Y, Z) respectively and $c = (c_1, ..., c_k)$ is the marginal of Z. We can then rewrite Eq. 1 as:

$$I(M) = \sum_{k=1}^{S} \frac{c_k}{N} \sum_{i=1}^{R} \sum_{j=1}^{C} \frac{n_{ijk}}{c_k} \log\left(\frac{c_k n_{ijk}}{a_{ik} b_{jk}}\right)$$

Where N is the sum of all elements in the table. We want to calculate its expectation over the set $\mathcal{M}$ of all possible tensors M that result in the same A and B

$$E\{I(M)|A,B\} = \sum_{M\in\mathcal{M}} \sum_{k=1}^{S} \frac{c_k}{N} \sum_{i=1}^{R} \sum_{j=1}^{C} \frac{n_{ijk}}{c_k} \log\left(\frac{c_k n_{ijk}}{a_{ik} b_{jk}}\right) P(M|A,B)$$

Here, we note that in our model of randomness, c is fixed, so we can rearrange following Nguyen et al.:

$$E\{I(M)|A,B\} = \sum_{k=1}^{S} \frac{c_k}{N} \sum_{i=1}^{R} \sum_{j=1}^{C} \sum_{n_{ijk}} \frac{n_{ijk}}{c_k} \log\left(\frac{c_k n_{ijk}}{a_{ik} b_{jk}}\right) P(M|n_{ijk}, A, B)$$

We also note that due to the lack of constrains on the (X, Y) contingency table, $P(M|n_{ijk}, A, B)$ only depends on elements a_{ik} and b_{jk} of A and B. Thus we have.

$$E\{I(M)|A,B\} = \sum_{k=1}^{S} \frac{c_k}{N} \sum_{i=1}^{R} \sum_{j=1}^{C} \sum_{n_{ijk}} \frac{n_{ijk}}{c_k} \log\left(\frac{c_k n_{ijk}}{a_{ik} b_{jk}}\right) P(M|n_{ijk}, a_{ik}, b_{jk})$$

And $P(M|n_{ijk}, a_{ik}, b_{jk})$ can be calculated [1] as:

$$P(M|n_{ijk}, a_{ik}, b_{jk}) = \frac{\binom{c_k}{n_{ijk}}\binom{c_k - n_{ijk}}{a_{ik} - n_{ijk}}\binom{c_k - a_{ik}}{b_{jk} - n_{ijk}}}{\binom{c_k}{a_{ik}}\binom{c_k}{b_{jk}}}$$

Adding that n_{ijk} can only take values between $\min(0, a_{ik} + bjk - c_k)$ and $\min(a_{ik}, b_{jk})$ we have the final expression for the expectation

$$E\{I(M|A,B)\} = \sum_{k=1}^{S} \frac{c_k}{N} \sum_{i=1}^{R} \sum_{j=1}^{C} \sum_{n_{ijk}=\min(0,a_{ik}+bjk-c_k)}^{\min(a_{ik},b_{jk})} \frac{n_{ijk}}{c_k} \log\left(\frac{c_k n_{ijk}}{a_{ik} b_{jk}}\right)$$
$$\frac{a_{ik}! b_{jk}! (c_k - a_{ik})! (c_k - b_{jk})!}{c_k! n_{ijk}! (a_{ik} - n_{ijk})! (b_{jk} - n_{ijk})! (c_k - a_{ik} - b_{jk} + n_{ijk})!}$$

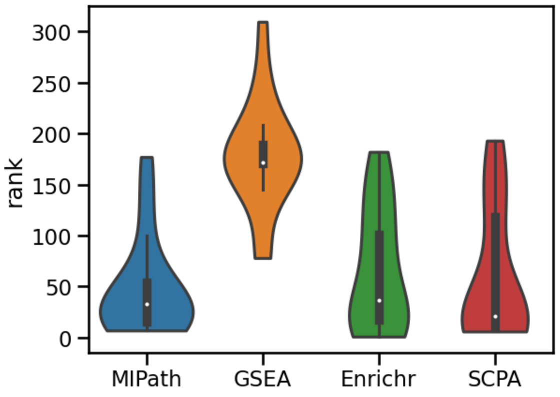

Figure 1: A visual representation of Table 2: A comparison of the performance between MIPath, GSEA, Enrichr and SCPA, based on the rank of selected target pathways for different datasets. For the comparison, we used the KEGG pathway with a total of 337 pathways. If two pathways had the same score, their ranks were averaged.

Reactome ID	Pathway name	MIPath score	Score accounting for treatment
R-MMU-425407	SLC-mediated transmembrane transport	0.2357	-6.74E-16
R-MMU-9006934	Signaling by Receptor Tyrosine Kinases	0.2148	-1.03E-16
R-MMU-449147	Signaling by Interleukins	0.2090	3.78E-16
R-MMU-166520	Signaling by NTRKs	0.2037	1.05E-15
R-MMU-382551	Transport of small molecules	0.2030	2.96E-16
R-MMU-448424	Interleukin-17 signaling	0.2004	4.82E-16
R-MMU-74160	Gene expression (Transcription)	0.2001	1.29E-16
R-MMU-1474244	Extracellular matrix organization	0.1990	-1.47E-16
R-MMU-425393	Transport of inorganic cations/anions and amino acids/oligopeptides	0.1986	1.39E-16
R-MMU-187037	Signaling by NTRK1 (TRKA)	0.1963	1.14E-16

Table 1: The top-scoring pathways associated with cell type on a stem cell reprogramming dataset [2], together with their score after accounting for the type of reprogramming treatment

References

[1] Xuan Vinh Nguyen, Julien Epps, and James Bailey. Information theoretic measures for clusterings comparison: is a correction for chance necessary? In *ICML*, 2009.

[2] Hannah T Stuart, Giuliano G Stirparo, Tim Lohoff, Lawrence E Bates, Masaki Kinoshita, Chee Y Lim, Elsa J Sousa, Katsiaryna Maskalenka, Aliaksandra Radzisheuskaya, Andrew A Malcolm, et al. Distinct molecular trajectories converge to induce naive pluripotency. *Cell Stem Cell*, 25(3):388–406, 2019.

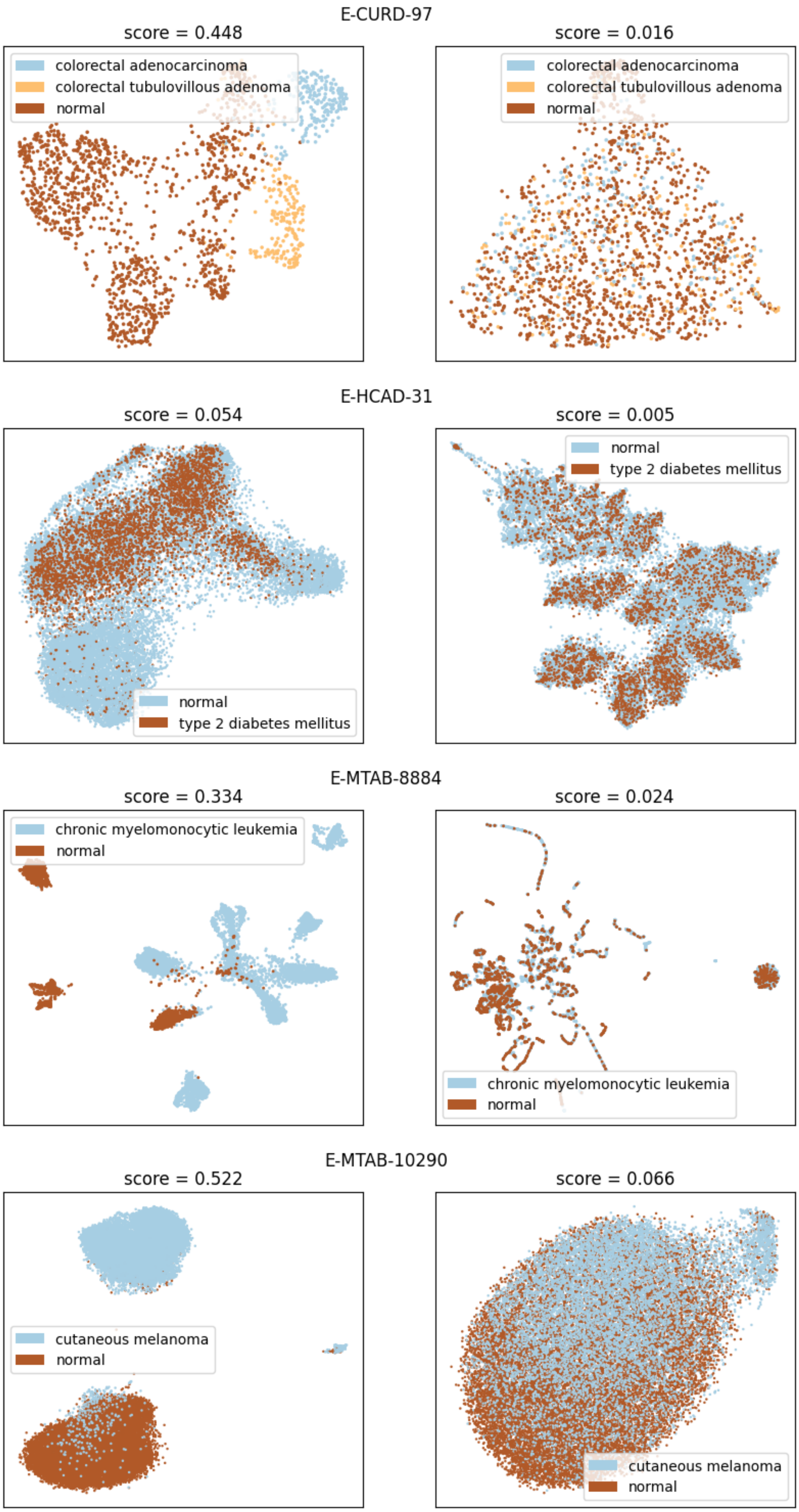

Figure 2: Visualization of cell separation for the highest scoring pathway and the median scoring pathway in multiple datasets.
